# Thermal effect of endoscopic thermal vapour ablation on the lung surface in human ex vivo tissue

**DOI:** 10.3109/02656736.2012.677932

**Published:** 2012-06-12

**Authors:** Erik Henne, Joseph C. Anderson, Robert Barry, Steven Kesten

**Affiliations:** Uptake Medical Corporation, Tustin, California, USA

**Keywords:** Cumulative injury, lung, thermal effect, vapour ablation

## Abstract

*Purpose*: An investigation of the thermal effect and the potential for injury at the lung surface following thermal vapour ablation (InterVapor), an energy-based method of achieving endoscopic lung volume reduction.

*Methods:* Heated water vapour was delivered to fifteen ex vivo human lungs using standard clinical procedure, and the thermal effect at the visceral pleura was monitored with an infrared camera. The time–temperature response was analysed mathematically to determine a cumulative injury quotient, which was compared to published thresholds.

*Results:* The cumulative injury quotients for all 71 treatments of ex vivo tissue were found to be below the threshold for first degree burn and no other markers of tissue injury at the lung surface were observed.

*Conclusion:* The safety profile for thermal vapour ablation is further supported by the demonstration that the thermal effect in a worst-case model is not expected to cause injury at the lung surface.

## Introduction

Lung volume reduction surgery (LVRS) improves lung ventilatory mechanics through a permanent alteration of lung anatomy. The National Emphysema Treatment Trial (NETT) conclusively demonstrated that LVRS results in long-term clinical improvements relative to a control group [1]. However, LVRS is characterised by significant short-term morbidity and a 5–8% 90-day mortality rate [1]. Endoscopic approaches to lung volume reduction have been developed with the goal of achieving some or all of the benefits of LVRS without the mortality and morbidity associated with major thoracic surgery.

One of the more promising endoscopic therapies (InterVapor) delivers thermal energy via heated water vapour to targeted regions of diseased hyperinflated lung. The initial localised inflammatory response is followed by a subsequent healing and repair process which, through fibrosis, permanently remodels lung tissue [2]. The contraction fibrosis and associated distal atelectasis results in reduction of tissue mass and air volume of the targeted lung regions. Treatment-induced lung volume reduction of hyper-inflated lung regions may result in increased elastic recoil by reducing the non-compliant areas of lung, decompressing areas of healthy lung allowing for alveolar recruitment, and improving the mechanical efficiency of the respiratory muscles. The therapy has been demonstrated to improve pulmonary function, exercise tolerance, and quality of life (based on St. George's Respiratory Questionnaire, a validated method of assessing health impairments in chronic obstructive pulmonary disease (COPD) patients) [3].

The dose of InterVapor energy is defined as calories per gram of lung tissue, which is calculated by software analysis of high resolution computed tomography (HRCT) scan. Energy doses too low may not achieve the necessary ablation and inflammatory response in the parenchyma to induce lung volume reduction, whereas doses too high may induce pleural adhesions or pneumothoraces [4].

These adverse effects occur at the lung surface, the visceral pleura. This study seeks to investigate the surface temperature change (thermal effect) at the pleura in human lung tissue after standard InterVapor doses to determine if the energy delivery and its thermal effect is capable of creating pleural injury.

## Methods

Ex vivo human lungs were chosen as a test model because emphysematous tissue can be obtained and it is possible to consistently monitor the thermal effect at the lung surface. The lungs were treated bilaterally in the upper lobes following a dosing and application procedure similar to that used clinically [3]. During and following treatment, the lung surface temperature was recorded by infrared camera. The relationship between surface temperature and time was used to calculate a cumulative injury quotient for each treatment, which was then compared to thresholds reported in the literature.

### Acquisition and preparation of human lungs

Non-transplantable human lungs excised *en bloc* were obtained from two tissue procurement agencies (International Institute for the Advancement of Medicine, Edison, NJ, USA and the National Human Tissue Resource Center, Philadelphia, PA, USA). The tissue requests were approved by an external feasibility committee for each agency. Both non-emphysematous and emphysematous lungs were included in the study. Emphysematous lungs were visually identified by the presence of hyperinflation, bullae, scarring, and deformities. No conditions were placed on the donor's age, sex, or height. Lungs were excluded from the study if they had significant fluid accumulation or did not inflate properly. During lung preparation, non-lung tissue, such as large blood vessels and adipose tissue, was removed from the lungs. Lungs were inflated to approximate functional residual capacity (FRC) (15 ±5cm H_2_O) and suspended from a modified trachea tube with an adapter for flexible broncho-scope. Lungs were studied within 48 h of receipt.

### Device description

The Uptake Medical InterVapor treatment system is comprised of a water vapour (steam) generator with a disposable catheter. The catheter tip with occlusion balloon is placed in the target airway via the working channel of a flexible fibre-optic bronchoscope. The proximal end of the catheter is attached to the vapour generator, which delivers a precise electronically controlled amount of vapour through the catheter to the target region of the lung. The water vapour is 100° C and 1 atm as it exits the catheter and dissipates energy to the lung tissue.

### Dosing

Vapour was delivered to the ex vivo lung following standard clinical treatment procedure [5] as closely as possible. In clinical application, vapour energy dose is quantified as calories per gram of tissue of the target region. The energy required to treat a lung segment is calculated by multiplying the dose (cal/g) by the segmental tissue mass (g). Tissue mass is calculated via software analysis of a lung HRCT scan [6]. With ex vivo tissue it is not feasible to perform an HRCT and calculate the segmental tissue mass prior to treatment. Instead, the required amount of energy to be applied to each segment of the ex vivo lung was calculated according to the following equation:


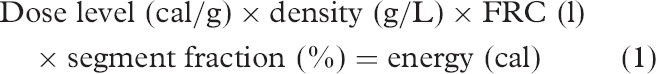


A dose level of 10 cal/g was tested because this is the maximum dose used in clinical trials [3]. Lung density was estimated based on published values. Healthy human lungs have a mean (SD) density of 322 (62) g/L at functional residual capacity (FRC) and emphysematous human lungs have a mean (SD) density of 209 (48) g/L [7]. Eleven of fifteen *en bloc* lungs tested had some degree of emphysema by visual inspection and 13 of 15 lungs had a history of smoking tobacco. For dose calculation it was assumed that all tested lungs had an in vivo density of 260 g/L. This density value is at the high end of the range for diseased lungs and lower end for healthy lungs. Using a density at the high end of the range (i.e. less disease) for lungs indicated for InterVapor ensured that delivered doses to the tissue would be at, or above, the target dose of 10 cal/g. FRC was the predicted value based on donor gender, age, and height [8]. Segment fraction is the ratio of the targeted sub-lobar volume to whole lung volume. Fractional contribution of lobes and sub-lobes was characterised by casting and dissecting 12 separate human lungs. Results are summarised in Table I. Using these values, the predicted tissue mass of the targeted segment was calculated by multiplying the density, predicted lung FRC, and segment fraction (Equation 1). Multiplying the target segment mass by the dose determined the energy required for InterVapor treatment.

**Table 1:** Lobar and sub-lobar segment fractions of the whole *en bloc* lung were used to estimate segment mass in the absence of CT analysis. Lobar fractions of the whole were determined by gas dilution [26]. To characterise sub-lobar segments (upper lobes only), the airways of twelve human ex vivo lungs were filled with silicone. The tissue was then dissolved with 2M KOH. The casted silicone airway branches of the upper lobes were dissected apart and weighed to determine the relative weight of each. The results were averaged and then multiplied by the fraction of the parent lobe to determine the fraction of the whole lung.

Lobar/Sub-Lobar Segment	Segment Fraction of Whole
Right Lung	52.50%
Right upper lobe	16.40%
RB1	5.72%
RB2	4.58%
RB3	6.10%
Right middle lobe	9.60%
Right lower lobe	26.50%
Left Lung	47.50%
Left upper lobe	24.00%
LB123	14.53%
LB12	6.35%
LB3*	8.19%
LB1	5.13%
LB2	3.24%
LB3+	6.16%
LB45	9.47%
LB4	4.85%
LB5	4.62%
Left lower lobe	23.50%

* LB3 value for anatomy with an LB12 apicoposterior configuration

+ LB3 value for anatomy with an LB1 and LB2 apicoposterior configuration

### Vapour application

**Figure 1:**
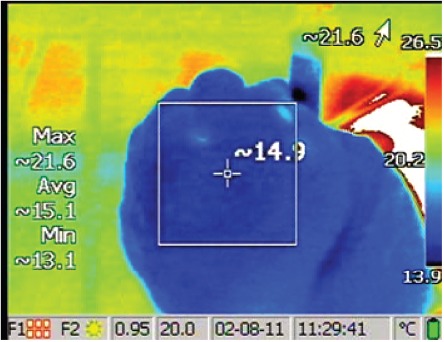
An example of surface temperature of the lung measured by infrared camera following a treatment. The values on the left describe the temperatures bounded by the square. The maximum lung surface temperature in this image is 21.6°C. The baseline temperature is subtracted from this maximum temperature to calculate the temperature change, examples in Figure 2.

A vapour dose of 10 cal/g was applied at the segmental or sub-segmental level to each of the upper lobes following the same method used clinically. Each lung required three to six treatments to completely treat the upper lobe, depending on segment size and airway access. To treat a targeted segment or sub-segment, the following steps were performed: (1) bronchoscope was inserted into the inflated lung and navigated to the airway for treatment, (2) catheter was advanced through the bronchoscope working channel, (3) catheter balloon was inflated to occlude the airway opening, (4) vapour application time (calculated based on the required energy) was entered into the generator, (5) catheter was attached to the generator, and (6) vapour was delivered to the target airway. During vapour application, and for at least 50 s following treatment, the surface temperature of the treated lung region was recorded with an infrared video camera (Figure 1). The procedure was repeated for each airway.

**Figure 2:**
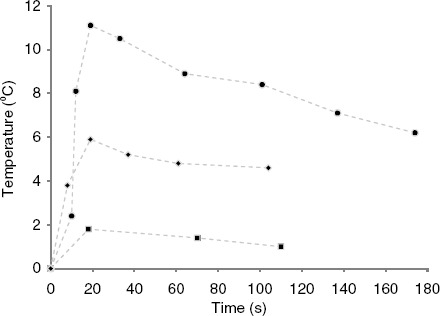
Plots of the discrete measurements of temperature change at the lung surface verses time after vapour delivery commencement (*t* = 0) for three representative temperature observations (high, medium, and low temperature change). The data was numerically integrated (Equation 2) using the trapezoid rule to determine the injury quotient. Data points were connected with dashed lines as a visual aid.

### IR video analysis

The surface temperature of the lung at the visceral pleura before vapour delivery was recorded and identified as the baseline temperature. The lung surface temperature of the region of interest was monitored during and following vapour delivery. The baseline temperature was subtracted from the maximum temperature of the region to calculate the temperature change. Between two and twelve (median was five) temperature change measurements were recorded per treatment, dependent on temperature change rate. Measurements were taken for at least 50 s following the start of a treatment or until the temperature decayed to 10° C above baseline, whichever came first.

### Data analysis

A cumulative injury integration of the time-temperature data collected was used to determine the net level of injury at the lung surface. The cumulative injury of the full thermal insult from baseline temperature at time 0 to the final temperature at time τ is obtained by integration of the injury rate, modelled using an Arrhenius expression:


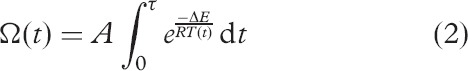


The cumulative degree of injury, Ω(*t*), was calculated numerically using Equation 2. Hyperaemia and focal epidermal necrosis (i.e. first degree burn) occurs when Ω = 0.53, and complete epidermal necrosis (i.e. third degree burn) occurs when Ω > 1 [9]. The universal gas constant, *R* = 8.314J/(mol K) and the temperature of tissue *T* (K) as a function of time *t* (s), are inputs to the expression. The frequency factor, *A*, and the activation energy for burn reaction, Δ*E*, have been empirically determined for many tissue types [10, 11]. However, no work has been done to characterise these parameters of thermal injury for pulmonary tissue. Because epidermis is the most thoroughly characterised tissue type and the most frequently cited [12], it was chosen for this analysis. In particular, the parameters identified by Henriques and Moritz [9, 14] were chosen (Table II) because they were among the most commonly cited in published literature [12].

**Table 2:** Kinetic parameters of cumulative injury (Equation 2) adopted from Henriques [9].

Property	Value	Units
Frequency factor, A	3.10×10^98^	s^−1^
Activation energy, E	627600	J/mol
Universal gas constant, R	8.3145	J/(mol K)
Cumulative injury, Ω, first degree	0.53	−
Cumulative injury, Ω, second degree	1.00	−

Because the treated ex vivo tissue was tested at room temperature, the average body temperature (310.15°K) was added to each surface temperature change measurement. Using the measured time-temperature data points as inputs, Equation 2 was numerically integrated using the trapezoid rule to determine the cumulative injury for each treatment.

For each treatment, the calculated cumulative injury was compared to the threshold for first degree burn (Ω = 0.53). While cellular changes may happen at lower levels [13], we theorise that the risk of pneumothorax begins at Ω = 0.53 or higher as this is the maximum exposure without the occurrence of necrosis [9]. Additional analysis of the cumulative injury was done by averaging the results for various sub-groups: comparison of emphysematous versus non-emphysematous tissue, comparison of segmental treatments verses sub-segmental treatments, and comparison by segment/sub-segment treated. These comparisons were done with a student's *t*-test and one-way variance analysis. Thermal isoeffective dose is another common method of reporting time-temperature data. Therefore, an additional *post hoc* analysis using the method for CEM43 (cumulative number of minutes at 43°C) was performed as outlined by Dewhirst et al. [10].

## Results

Fifteen bilateral lungs were included in the study. All segments of both upper lobes were treated except for regions with obvious lacerations, which occasionally occur during tissue procurement or preparation and affect thermal distribution. A total of 79 treatments were delivered to ex vivo tissue. Eight of these treatments were omitted from analysis because they were either not delivered according to protocol (failure to maintain catheter position or deliver correct treatment time) or were in regions with previously undiscovered lacerations.

The mean (SD) vapour application time was 6.1 (1.5) s. Clinically, and in this study, InterVapor treatment is limited to a range of 3–10 s for quality control and safety. Vapour is delivered at the segmental level unless factors such as segment size, airway geometry, or airway diameter make a sub-segmental treatment preferable. The same factors were considered during the ex vivo study. Of the 71 successful treatments included in analysis, 55 (77%) were at the segmental level and 16 (23%) were at the sub-segmental level.

The temperature change at the lung surface versus time for three representative sets of measurements is plotted in Figure 2. The mean (SD) peak temperature change of all vapour deliveries was 4.8°C (3.4). The location of the thermal effect at the lung surface corresponded with the airway being treated. No lung inflation, tissue ablation, collagen shrinkage, or other tissue damage was observed at the surface of the lung or within view of the scope. This is expected, as the effects of InterVapor manifest over a number of weeks after treatment [5].

Equation 2 was used to calculate the cumulative injury at the lung surface, Ω, which ranged from 0 to 0.076 with a mean (SD) of 0.0025 (0.0098) and a median of 0.0001 for each treatment. Eleven of the 15 lungs showed signs of emphysema, the mean (SD) Ω in this group was 0.0029 (0.0109). The mean (SD) Ω of the remaining lungs with no visual evidence of emphysema was 0.0013 (0.0029) (*p* = 0.33). The mean (SD) Ω of treatments at the segmental level was 0.0031 (0.0110) and the mean (SD) of treatments at the sub-segmental level was 0.0008 (0.0022) (*p* = 0.42). A one-way analysis of variance was done to compare the mean Ω of each segment and sub-segment tested and no significant difference between treatment locations was found (*p* = 0.21). A summary of these statistical comparisons are shown in Table III and the mean Ω of treatments to each lung are summarised in Table IV.

**Table III:** Summary of statistical comparisons between the average cumulative injury (Ω) for various subgroups using a Student's *t*-test.

		Ω		
			
	Number of treatments	Average (SD)		*p*
Treatments in lungs with visual evidence of emphysema Treatments in lungs with no visual evidence of emphysema	56 56	0.0029 0.0013	(0.0109) (0.0029)	0.33
Treatments at the segmental level Treatments at the sub-segmental level	55 16	0.0031 0.0008	(0.0110) (0.0022)	0.42
All treatments	71	0.0025	(0.0098)	

**Table IV:** Average cumulative injury (Ω) following vapour ablation to each lung. The median cumulative injury of all treatments was 0.0001.

Lung ID	Visual evidence of emphysema	Number of treatments	Ω Average (SD)	
110207-A		2	0.0021	(0.00221)
110208-A	X	6	0.0010	(0.00227)
110211-A	X	3	0.0000	(0.00001)
110211-B	X	3	0.0032	(0.00461)
110301-A	X	7	0.0001	(0.00011)
110301-B	X	4	0.0001	(0.00004)
110301-D		5	0.0003	(0.00031)
110315-A		5	0.0027	(0.00469)
110318-A		3	0.0000	(0.00001)
110325-A	X	4	0.0007	(0.00129)
110502-A	X	8	0.0000	(0.00002)
110502-B	X	4	0.0107	(0.01307)
110511-A	X	6	0.0022	(0.00458)
110520-A	X	6	0.0129	(0.03083)
110525-A	X	5	0.0016	(0.00347)
Average		4.7	0.0025	(0.00450)

Another common method of reporting time-temperature data is thermal isoeffective dose. The method outline by Dewhirst et al. [10] was used to calculate the cumulative number of equivalent minutes at 43°C (CEM43) for each treatment at the visceral pleura. For the temperature term, the average of the maximum temperature of the region over time was used. The mean (SD) CEM43 of all treatments was 1.43 (6.34).

## Discussion

Endoscopic thermal vapour ablation (InterVapor) has recently been approved in Europe for treatment of heterogeneous severe emphysema. Data indicate that InterVapor treatment improves lung function, exercise tolerance, and quality of life with a favourable benefit to risk profile [3]. A previous animal study suggested unintended injury to the lung surface may occur, which could cause pleural adhesions, if thermal energy is applied at high doses [4]. It was theorised that in fragile emphysematous tissue this could lead to pneumothorax. The current study was designed to determine the potential for injury at the lung surface in an ex vivo human lung model with and without emphysema. The current investigation found all the temperature changes at the lung surface following InterVapor treatments to be within a safe range (Ω < 0.53, threshold for first degree burn).

A human ex vivo lung model was considered the optimal approach for this investigation because viable animal models, such as sheep and canine, differ significantly from humans in terms of airway anatomy (i.e. monopodial verses dichotomy branching). In addition, a papain-induced emphysema model in animals, while a reasonable approach, still differs from tobacco smoke-induced emphysema developing over years to decades in humans. Finally, an ex vivo test set-up was more consistent, controllable, and allowed for large surface areas to be observed.

For some investigations of the effect of thermal ablation in the lung, ex vivo tissue is not a suitable model because the differences are too large [15]. However, because the primary aim of this study was to examine the potential for injury at the lung surface due to vapour, human ex vivo tissue represents a suitable ‘worst case’ model as compared to in vivo for four reasons. First, the ex vivo tissue is exsanguinated. Blood perfusion in living human lung tissue has been observed to transport heat away after being submitted to thermal ablation [16, 17]. This effect is absent in the ex vivo model. Second, the vapour dose in this study was calculated assuming the tissue was perfused because the density estimation was taken from measurements of intact lungs. Lung mass is approximately 60% blood and 40% extravascular tissue [18]. As a result, delivered energy to ex vivo tissue with minimal blood present is up to 150% higher per gram than it would have been in vivo. Third, the current study investigated the thermal effect at the lung surface. In the ex vivo model the lung surface is in contact with air while the in vivo lung is in contact with adjacent tissue. Assuming adjacent tissue has the same thermal conductivity as water; thermal conductivity to adjacent tissue is over 23 times higher than air thereby more rapidly cooling the tissue [19]. Fourth, the respiratory airflow present in intact lungs, which is not present in ex vivo lungs, removes heat. It has been shown that lesions from thermal ablation are larger in non-ventilated lung tissue as compared to ventilated tissue [22]. In clinical usage the airway is occluded with a balloon during vapour delivery, which is 3 to 10 s in duration. The balloon is then deflated within 15 seconds of vapour delivery completion allowing fresh air from mechanical ventilation to enter the segment shortly after treatment. In this study, temperatures were measured for at least 50 s following treatment and the balloon occluded the airway the entire time. These four significant contributors to cooling in living tissue are not present in ex vivo tissue and suggest that any thermal effect observed in ex vivo lung tissue due to vapour ablation would be higher than the corresponding effect in vivo.

The primary inherent limitation of the study is the difficulty in accurately estimating sub-lobar segment mass in ex vivo tissue, which relates directly to the amount of energy delivered during treatment. A density at the high end of the expected range was chosen for calculation to ensure that most doses would be above the target of 10 cal/g. The lack of empirical data characterising thermal injury of pulmonary tissue is also a limitation. Quantitative injury in this study was calculated using the commonly cited parameters for epidermis identified by Henriques and Moritz [9]. More conservative parameters for epidermis from Mehta et al. [20] and Weaver et al. [21] were also considered and similar results were found. For these parameters only one and two of the 71 treatments respectively created a thermal effect larger than the 0.53 injury threshold. Considering the rapid development in thermal ablation of lung tissue technology from laser [15] to lung perfusion [23] to radio frequency [17, 24, 25] to vapour, a characterisation of the frequency factor and activation energy for thermal injury of pulmonary tissue is needed. Characterisation of thermal injury parameters for pulmonary tissue would allow for more precise analysis. This would be useful for analysis of higher vapour ablation doses, which would result in higher temperatures and cumulative injury with a smaller safety margin.

## Conclusion

Endoscopic thermal vapour ablation is designed to produce a thermal effect resulting in a controlled injury of the airways and parenchyma within the targeted regions of diseased hyperinflated lung. A thermal effect at the lung surface, however, could induce pleural adhesions or pneumothoraces. To characterise the thermal effect and the potential for injury at the lung surface, the temperature change of 71 treatments to ex vivo human tissue was analysed. The results of this study found the cumulative injury quotient at the lung surface remained well within a safe range (<0.53) for each treatment despite the fact that ex vivo tissue lacks many of the cooling mechanisms present in vivo, including blood circulation, ventilation, and conduction to adjacent tissue. The median injury coefficient, at 0.0001 is well below the 0.53 threshold. Additionally, no significant difference in injury coefficient was found between treatments to emphysematous and healthy tissue and between treatments at the segmental and sub-segmental level. While InterVapor is designed to produce a controlled injury near the targeted airway, these results demonstrate that treatment in the dose range of 10 cal/g and lower is not expected to be associated with injury at the lung surface. This is consistent with pre-clinical [2, 4] and clinical findings [3]. In addition to no other visible changes to lung tissue, the results suggest that there remains a margin of safety at a dose of 10 cal/g. In conclusion, the present study provides clinically relevant data from explanted human lungs supporting the safety profile of endoscopic thermal vapour ablation at the dose that is currently approved for use in patients with severe emphysema.
